# Indigenous and Tribal Peoples Data Governance in Health Research: A Systematic Review

**DOI:** 10.3390/ijerph181910318

**Published:** 2021-09-30

**Authors:** Kalinda E. Griffiths, Jessica Blain, Claire M. Vajdic, Louisa Jorm

**Affiliations:** 1 Centre for Big Data Research in Health, University of New South Wales, Sydney, NSW 2052, Australia; j.blain@unsw.edu.au (J.B.); claire.vajdic@unsw.edu.au (C.M.V.); l.jorm@unsw.edu.au (L.J.); 2 Wellbeing and Preventable Chronic Diseases Division, Menzies School of Health Research, Tiwi, NT 0812, Australia; 3 Centre for Health Equity, University of Melbourne, Parkville, VIC 3010, Australia

**Keywords:** indigenous health, data governance, data, Aboriginal and torres strait islander health, health policy, equity

## Abstract

There is increasing potential to improve the research and reporting on the health and wellbeing of Indigenous and Tribal peoples through the collection and (re)use of population-level data. As the data economy grows and the value of data increases, the optimization of data pertaining to Indigenous peoples requires governance that defines who makes decisions on behalf of whom and how these data can and should be used. An international a priori PROSPERO (#CRD42020170033) systematic review was undertaken to examine the health research literature to (1) identify, describe, and synthesize definitions and principles; (2) identify and describe data governance frameworks; and (3) identify, describe, and synthesize processes, policies and practices used in Indigenous Data Governance (ID-GOV). Sixty-eight articles were included in the review that found five components that require consideration in the governance of health research data pertaining to Indigenous people. This included (1) Indigenous governance; (2) institutional ethics; (3) socio-political dynamics; (4) data management and data stewardship; and (5) overarching influences. This review provides the first systematic international review of ID-GOV that could potentially be used in a range of governance strategies moving forward in health research.

## 1. Introduction

Data underpin our ability to optimize systems, services, and policy in society. However, research regarding the governance of Indigenous and Tribal Peoples (hereafter respectfully Indigenous) health research data is nascent. Specifically, the term ‘data governance’ is conceptualized within western constructs of institutional and information technology governance [1]. This has potential implications for how Indigenous Data Governance (ID-GOV) is actualized. Importantly, the global movement of Indigenous Data Sovereignty (ID-SOV), whereby Indigenous peoples have the right to exercise authority and govern the affairs of the use of Indigenous data that reflects Indigenous peoples interests and aspirations [2], has become a necessary consideration in how data pertaining to Indigenous peoples around the globe are collected, owned and used. As the data era has encroached and exploded, so too has the value of data, both as a tangible asset, but also as a tool to drive change in response to health, social, economic, ecological, and cultural needs. To further advance this developing area of research, this systematic literature review provides a comprehensive overview of the global literature describing ID-GOV in health research.

Discussions on the development of data and information pertaining to Indigenous peoples has been occurring for over half a century. Initial issues regarding Indigenous data were acknowledged and aimed to be addressed internationally through the United Nations International Labour Organization Conventions No. 107 (1957) and No. 169 (1989) [3,4]. The 2007 United Nations Declaration on the Rights of Indigenous People (UNDRIP) emphasizes the rights of Indigenous peoples to live in dignity, to maintain and strengthen Indigenous institutions, cultures and traditions, and to pursue self-determined development of Indigenous needs and aspirations [5]. This includes the quality and usability of Indigenous data as well as how it can best serve the needs and aspirations of Indigenous peoples. Central to the data collected about Indigenous peoples has been the issue of identification, which is critical for the visibility of Indigenous people within nations. There have been years of international efforts from Indigenous and non-Indigenous scholars, public servants, and community members to improve the identification of Indigenous peoples in national data collections [6]. However, whether Indigenous data is collected appropriately, which Indigenous data are collected and the resultant narrative arising from those data is impacted by the systems that oversee them [2]. Importantly, considerations regarding how the rights of Indigenous people can be met through data have also raised discussions about data collections and the presentation of data as well as the consistency of Indigenous reporting in national data collections [7].

Health research data within the Indigenous context pertains to all data collected and (re)used for the purposes of health and wellness. For Indigenous peoples, this includes Indigenous understandings of health and wellness which moves beyond individual biomedical understandings of health to also include the health and wellness of the community and the environment over time. While there are an extensive range of definitions that describe Indigenous understandings of health around the globe, the inclusion of community health and ecological health appear consistently. This means that the scope of data that pertains to Indigenous health will be broader than what is typically seen within western biomedical constructs of health. In terms of measurement, this will include those factors that are considered important to Indigenous peoples and their communities. Specifically, this involves the identification and measurement of the social, cultural and economic and ecological determinants of health as well as the historical and continuing impacts of colonization upon the health and wellness of Indigenous people [8]. These measures are necessary to identify gaps and support progress in addressing disparities within nations.

Advancing health research that supports equity requires accurate and appropriate data. There is a legacy of Indigenous invisibility and inappropriate collections and uses of data pertaining to Indigenous peoples. This has generated calls internationally for statistical agencies to guarantee the visibility of Indigenous peoples in their national data [6], as well as appeals to nations to support the development of measures and metrics that reflect the needs and aspirations of Indigenous peoples [5]. The enactment of these requirements, however, has needed Indigenous governance.

Developments in ID-SOV have progressively been occurring across a range of nations, primarily within Canada, Aotearoa/New Zealand, the United States and Australia since 2016 [2]. ID-GOV guidelines and recommendations for the implementation of ID-SOV in practice are now also being developed globally [9]. This in turn requires support for the recognition of individual and collective human rights as well as sovereign rights. Specific to data, it is necessary for the inclusion of overarching international human rights mechanisms such as the UNDRIP as well as local level cultural authority to enable diversity within decision making processes relating to data [5]. Generally, data governance refers to what decisions must be made to ensure the effective management and use of data, who should make these decisions as well as how these decisions will be made [1].

There has been an ongoing argument for appropriate, accurate and quality population level health data that reflects Indigenous understandings as well as historical and contemporary experiences of Indigenous people [6]. There has also been considered international scholarship to identify and understand the historical, social, cultural, ecological and economic contexts that can impact the health and wellbeing of Indigenous people around the globe [10]. The interest and importance of data that appropriately reflect Indigenous peoples and support sovereign rights is also displayed in the growing scholarship of ID-SOV and ID-GOV. This centralizes the question, “who makes decisions on behalf of whom, when it comes to Indigenous data?” Within institutional research frameworks, the importance of recognizing the complex and diverse experiences of Indigenous peoples can only be appropriately described by Indigenous peoples [11]. Globally, advancements in data have set the pace of the development of data governance frameworks, models and processes that have arisen largely out of need. To describe this growth and development, this review aims to examine the international literature describing ID-GOV in health research to:

a. Identify, describe, and synthesize definitions and principles used in Indigenous peoples data governance in health research and reporting.
b. Identify and describe data governance frameworks in the health research literature that include Indigenous peoples data.
c. Identify, describe, and synthesize processes, policies and practices used in Indigenous peoples data governance in research and reporting of health and wellbeing.

## 2. Materials and Methods

This review has arisen from many years of community and academic discussions about rights regarding Indigenous data. This systematic review, which is part of a larger body of research, is led by Yawuru woman, Kalinda Griffiths under the guidance of Aboriginal community and academic leaders in Australia, the United States, Aotearoa/New Zealand, and Canada, primarily through the International Group for Indigenous Health Measurement. Additionally, it is also supported by non-Indigenous academic leaders in data science, epidemiology, and health research. The other authors on this paper are non-Indigenous, academics and non-academics, working closely with Indigenous researchers and communities to support community priorities. This partnership aims to continue collaborative efforts in Indigenous prioritized research as well as to build the capacity of emerging Indigenous and non-Indigenous researchers working in Indigenous spaces.

An international *a-priori* PROSPERO (#CRD42020170033) systematic review was conducted and reported in accordance with PRISMA guidelines [12]. Qualitative meta-aggregation, whereby the synthesis of thematic findings across a range of methodologies, was applied to abstracted data [13].

### 2.1. Inclusion/Exclusion Criteria

Included publications were published in English from 1 January 2001 to 1 April 2021. There were no limitations on the category of research (qualitative vs quantitative) nor on the study designs. Published peer-reviewed publications, books, technical reports, and guidelines were included. Due to content requirement for data abstraction, conference proceedings without a full publication as well as letters were excluded. 

Publications that described the governance of data within Indigenous people’s population level health and wellbeing research were included. Publications that were not specific to health research, did not include discussions regarding data and did not include Indigenous peoples were excluded. 

### 2.2. Search Strategy

Sources used to identify publications included bibliographic databases (BioMed Central, PubMed, Scopus), reference lists of eligible publications, search engines (Google and Google Scholar) as well as expert input about relevant publications from investigators and external experts. Relevant database searches were conducted until 1 June 2020 and were then re-run prior to the final analyses (1 April 2021).

There were three concepts used in this search strategy. Specifically, **(1) ‘Indigenous and Tribal Peoples’** [(indigenous), (aborigin* AND torres strait islander*), (aborigin* OR torres strait islander*), (first nation*), (metis), (alaskan native*), (american indian*), (maori*), (saami*), (inuit*), and (tribal people*)]; **(2) ‘data governance’** [(data AND govern*), (data AND manage*), (information AND manage*), (data AND legislat*), (data and regulat*), (data AND polic*), (data AND procedure*), (data AND process*), (data AND secur*), (data AND integrity), (data AND steward*), (data AND custodian*), (data AND asset), (data AND sovereign*), (data AND framework*), (data AND compliance), (data AND accountab*), (data AND access), (data AND trust), (data AND ethics), (data AND risk), (data AND disclosure), (data AND reporting), (data AND safe), (data AND confidential*), and (data AND privacy)]; and **(3) ‘health research’** [(health AND research), (medical AND research), (health AND stud*), (medical AND stud*)]. Following pilot searches, we removed the following search terms: (data AND polic*), (data AND procedure*), (data AND process*), (data AND secur*), (data AND integrity), (data AND compliance), (data AND access), (data AND trust), and (data AND confidential*) as they introduced many irrelevant records. The final applied search strategy can be found in Appendix A.

### 2.3. Search Strategy and Data Extraction

**Figure 1:**
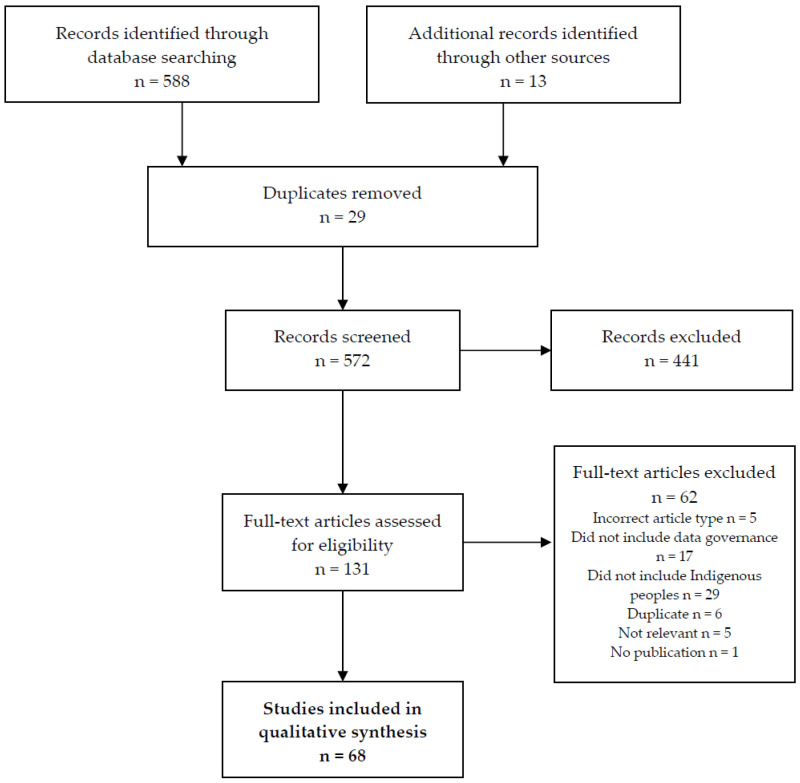
PRISMA screening process to assess Indigenous Data Governance in health research.

The 601 titles and abstracts retrieved through electronic searching were downloaded onto Covidence [14]. 131 publications were independently assessed for eligibility by at least two reviewers (K.E.G., J.B., Research Assistant). Disagreements on inclusion were considered by a third reviewer (C.M.V.). Of these, 68 publications were included for the qualitative synthesis. The main reason for exclusion was that Indigenous peoples were not reported on. See Figure 1 for PRISMA screening process.

Data abstraction was conducted by two reviewers (K.E.G. & J.B.). We used thematic analysis to identify and categorize elements that were found to be relevant to ID-GOV across a range of domains. ID-GOV domains were selected through initial review for the development of an *a-priori* data abstraction tool. The domains were ID-GOV definitions, data governance principles and processes, Indigenous engagement, and decision-making processes, as well as policies and practices identified as relevant to the governance of Indigenous data.

## 3. Results

### 3.1. Overview

Almost 90% of the included 68 publications were published in the last 8 years (*n* = 61). Countries/regions of focus were primarily across the nations of Canada (*n* = 23), the United States (*n* = 17), Australia (*n* = 12) and Aotearoa/New Zealand (*n* = 11). Over half of the publications were either editorials/essays (*n* = 21) or reports (*n* = 17). See Table 1.

### 3.2. Data Governance Definitions in Indigenous Peoples Health Research

Fourteen definitions of data governance were described in the articles. Seven articles defined data governance in relation to mechanisms for the management and stewardship of data, including reference to processes, protocols, policies, practices, standards, frameworks, and infrastructure [15,16,17,18,19,20,21,22]. For example, “[Data management] refers to the policies, protocols, and practices related to data collection; analysis and interpretation; storage and security; sharing; withdrawal and disposal; Return of results to participants and dissemination of results to the broader public” [15]. Additionally, management approaches were also at the community-level. For example, “[Community-level governance] of research refers to the use of community-based mechanisms for guiding and regulating research” [15]. Furthermore, data governance was delineated as a western construct as defined by the United Nations Development Program in Carroll’s articles as “the system of values, policies and institutions by which a society manages it economic, political and social affairs through interactions within and among the state, civil society and private sector” [22].

Sixteen articles defined ID-GOV or the governance of Indigenous data [2,15,16,17,18,19,20,21,23,24,25,26,27,28,29,30]. Of these, seven articles referred to the inherent rights of Indigenous peoples in data governance [2,18,21,22,23,24,30], including specific references to Indigenous data governance supporting self-determination [2,18,21,22,23] and nation rebuilding (whereby nation rebuilding includes processes of reclamation of self-rule and increased self-determination) [22]. Five articles explicitly mention Indigenous peoples’ control over data in the definitions [2,16,18,20,22,24,26]. Five articles mention Indigenous values and/or understandings in definitions [2,18,21,23,26]. ID-GOV was also explicitly described as the operationalization of ID-SOV [21]. Definitions did not always include a description of ID-GOV policies, processes, or practices. Two publications specified that ID-SOV is enacted through ID-GOV [21,22]. One publication stated that their governance framework synthesizes key aspirations for ID-SOV [31].

Research governance and data governance were sometimes intertwined with recognition that overarching research systems will result in data governance processes within organisations. Two articles define data/research governance in relation to “inclusion” of Indigenous peoples or partnerships between Indigenous communities and research institutions [18,23]. For example, “(Research) Governance relates to partnerships between the research institution(s) and Indigenous organization(s) to recognize the centrality of Indigenous self-determination and leadership in research conduct and to provide an accountability mechanism by which the host research institution aims to meet the principles, expectations, priorities, and values of Indigenous research stakeholder(s)” [23].

### 3.3. Data Governance Frameworks and Principles in Indigenous Peoples Health Research

Data governance frameworks provide the mandates, practices, processes, and roles by which data should be governed. Seven data governance frameworks were identified within the literature. Table 2 provides an overview of specific data governance frameworks along with their principles and domains. This included 5 global frameworks (Five Safes Framework, Global Alliance for Genomics and Health’s Framework for Responsible Sharing of Genomic and Health-Related Data, the CONSIDER statement, the CARE principles, and the FAIR principles) [9,23,32,33,34]. Two of the frameworks were explicitly focused on Indigenous data (the CONSIDER statement, the CARE principles) [9,23].

The principles and/or domains of the data governance frameworks are provided to identify the key components of the frameworks. For this review, principles are defined as foundational standards in the use of data and domains are fields or activities that pertain to data. Based on this, there were three categories identified. This includes (1) General principles, which are principles incorporated into data governance frameworks that can be used across all populations; (2) Indigenous principles, which are principles that are explicit to Indigenous populations; and (3) Action domains, which are prescriptive actions for individuals using data. These are not mutually exclusive meaning that principles may also incorporate actions.

Two ID-GOV frameworks stated they were underpinned by ID-SOV principles (the CONSIDER statement and the CARE principles) [9,23]. Twenty-five articles describe ID-SOV [2,9,16,17,18,22,23,24,25,27,28,29,35,36,37,38,39,40,41,42]. These articles define ID-SOV as affirming the right for Indigenous peoples to control the ownership, access, collection, management and/or use/reuse of Indigenous data as first defined in Kukutai and Taylor’s 2016 publication [2]. Descriptions of ID-SOV mention the aim to ‘protect collective interests’ [37] and support “collective benefit” as described in the CARE principles and referred to in Walter and Carroll’s articles [9,21,22]. ID-SOV protects and develops “cultural heritage, traditional knowledge and traditional cultural expressions” and Indigenous peoples’ “right to maintain, control, protect and develop their intellectual property over these” [39]. The demand for transparency of research practices was also described [23], which is also noted in the data governance frameworks.

### 3.4. Policies, Proceses and Practices to Advance Indigenous Data Governance in Indigenous Peoples Health Research

All 68 articles described the governance of data with at least one reference to Indigenous peoples [2,15,16,17,18,19,20,21,22,23,24,25,26,27,28,29,30,31,35,36,37,38,39,40,41,42,43,44,45,46,47,48,49,50,51,52,53,54,55,56,57,58,59,60,61,62,63,64,65,66,67,68,69,70,71,72,73,74,75,76,77,78,79,80,81,82,83,84]. Of these, 20 publications discussed policies, processes, and practices of Indigenous data in depth [2,15,17,20,21,22,25,26,30,31,37,38,44,45,46,49,50,57,72,81]. Both the United States and collaborative global efforts had seven publications each, where three of these articles were specific to Canada, one to Australia, two to Aotearoa/New Zealand. Table 3 provides an overview of policies, processes and practices that could potentially be used to advance ID-GOV. It specifies the primary themes in the publications, identifies the barriers and challenges mentioned, and the considerations that were highlighted. Those articles not included mentioned data governance and Indigenous peoples within contexts, however, did not provide further details.

The thematic synthesis from the assessment of governance policies, processes and practices in Indigenous health research was broad. From the synthesis, five categories arose in which policies, processes, and practices of ID-GOV fit (See Appendix B). These categories were found to be interrelated, context specific and active. They were shown to be enacted through data or research frameworks, protocols, policy, and principles. The categories are:

1. Indigenous governance.
2. Institutional ethics.
3. Socio-political dynamics.
4. Data management and data stewardship.
5. Overarching influences.

These are expanded further in the next section.

**Table 2:** Data governance frameworks identified in the systematic review of Indigenous Data Governance in health research.

Data Governance Frameworks	Overview	Principles/Domains
Five Safes [2,21]	The Five Safes is an approach to thinking about, assessing, and managing risks associated with data sharing and release.	Safe people—researchers can be trusted to use data appropriately and follow procedures. Safe projects—the project has a statistical purpose and is in the public interest. Safe settings—security arrangements prevent unauthorised access to the data. Safe data—the data inherently limit the risk of disclosure. Safe output—the statistics produced do not contain any disclosing results.
OCAP^®^ [2,15,16,17,18,19,20,21,24,26,30,37,39,49,52,56,61,66,67,72,73,77]	Establish how First Nations’ data, information, and cultural knowledge should be collected, accessed, used, and shared.	Ownership: The notion of ownership refers to the relationship of a First Nations community to its cultural knowledge/data/information. The principle states that a community or group owns information collectively in the same way that an individual owns their personal information. Ownership is distinct from stewardship. The stewardship or custodianship of data or information by an institution that is accountable to the group is a mechanism through which ownership may be maintained. Control: The principle of “control” asserts that First Nations people, their communities and representative bodies must control how information about them is collected, used and disclosed. The element of control extends to all aspects of information management, from collection of data to the use, disclosure and ultimate destruction of data. Access: First Nations must have access to information and data about themselves and their communities, regardless of where it is held. The principle also refers to the right of First Nations communities and organizations to manage and make decisions regarding who can access their collective information. Possession: While “ownership” identifies the relationship between a people and their data, possession reflects the state of stewardship of data. First Nations possession puts data within First Nations’ jurisdiction and, therefore, within First Nations’ control. Possession is the mechanism by which to assert and protect ownership and control. First Nations generally exercise little or no control over data that are in the possession of others, particularly other governments.
Global Alliance for Genomics and Health’s Framework for Responsible Sharing of Genomic and Health-Related Data [43]	Provides guidance for the responsible sharing of human genomic and health-related data, including personal health data and other types of data that may have predictive power in relation to health.	− Respect Individuals, Families and Communities. − Advance Research and Scientific Knowledge. − Promote Health, Wellbeing and the Fair Distribution of Benefits. − Foster Trust, Integrity and Reciprocity.
CONSolIDated critERtia for strengthening the reporting of health research involving Indigenous Peoples (CONSIDER) statement. (Global) [23]	Provides a checklist for the reporting of health research involving Indigenous peoples to strengthen research praxis and advance Indigenous health outcomes.	(i) Governance (ii) Relationships (iii) Prioritization (iv) Methodologies (v) Participation (vi) Capacity (vii) Analysis and findings (viii) Dissemination
CARE principles (Global) [21]	Are people and purpose-oriented, reflecting the crucial role of data in advancing Indigenous innovation and self-determination. They complement the existing FAIR principles encouraging open and other data movements to consider both people and purpose in their advocacy and pursuits.	Collective benefits: Data ecosystems shall be designed and function in ways that enable Indigenous peoples to derive benefit from the data. Authority to control: Indigenous peoples’ rights and interests in Indigenous data must be recognized and their authority to control such data be empowered. Indigenous data governance enables Indigenous peoples and governing bodies to determine how Indigenous peoples, as well as Indigenous lands, territories, resources, knowledges and geographical indicators, are represented and identified within data. Responsibility: Those working with Indigenous data have a responsibility to share how those data are used to support Indigenous peoples’ self-determination and collective benefit. Accountability requires meaningful and openly available evidence of these efforts and the benefits accruing to Indigenous peoples. Ethics: Indigenous peoples’ rights and wellbeing should be the primary concern at all stages of the data life cycle and across the data ecosystem.
FAIR principles (Global) [37]	Aim to help create, share and re-use quality, valuable, and responsible data.	Findable: Resource and its metadata are easy to find by both, humans and computer systems. Accessible: Resource and metadata are stored for the long term such that they can be easily accessed and downloaded or locally used by humans and ideally also machines using standard communication protocols. Interoperable: Metadata should be ready to be exchanged, interpreted and combined in a (semi)automated way with other data sets by humans as well as computer systems. Reusable: Data and metadata are sufficiently well described to allow data to be reused in future research, allowing for integration with other compatible data sources.
Integrated Data Infrastructure (IDI) Ngā Tikanga Paihere framework [37]	The framework guides the appropriate use of microdata in the IDI, with a focus on how data about Māori and other under-represented sub-groups are used for research purposes. This framework was underpinned by the Five Safes framework.	Pūkenga (knowledge and expertise): Researchers can demonstrate an awareness of and intention to work with data in culturally appropriate ways. Pono (accountability and transparency): Level of accountability to communities of interest is explained and there is community support for the research. Wānanga (organizations): Institutions have established systems, policies and procedures to support culturally appropriate practices when working with data. Wairua (community good): Community objectives align with project research objectives and any potential harm to these groups is considered. Noa (benefit and opportunity): Data are readily accessible and there is demonstrated awareness of the impact on communities of interest.

**Table 3:** Policies, processes and practices to advance ID-GOV.

Ref/Year	Title	Policies, Processes, Practices as Necessary to ID-GOV	Topic/s	Challenges and Barriers to ID-GOV	Considerations for the Advancement of ID-GOV
Canada					
[45]/2001	Building Capacity in Applied Population Health Research	Develop population health that is consistent with Ownership, Control and Accessible (OCA). Shared leadership, power, and decision making from design to dissemination. Respect. Resolution (through the Assembly of Manitoba Chiefs) to build research capacity and to extend further First Nation control over the health care systems in their communities.	Health research.	Over-emphasis if pathologizing discourses in Aboriginal health research. Institutional barriers and ownership issues to effective research dissemination. Lacking technical and analytical skills to make information relevant to community health needs and interests.	Build human capital in population health research. Build social capital through the OCA principle. Research institutions, academics, and governments to develop agreements that respect First Nation determination. Build First Nation research capacity. Build partnerships.
[44]/2006	The Manitoba First Nations Centre for Aboriginal Health Research: knowledge translation with Indigenous communities	Participation in the research process from the start. Degree of control or ownership over the research process. Engagement. Trust.	Knowledge translation in research.	The need is greater than the capacity. Ownership and control of research data.	Trust and partnerships.
[30]/2014	Barriers and Levers for the Implementation of OCAP	If there are gaps in legislation, then appropriate tools should be used to apply OCAP^®^. Preserve ownership and other IP rights. Making OCAP^®^ tools available for use by communities.	Data. Law. Knowledge and capacity.	Identifying applicable laws. Legislative obstacles. Lack of available information. Unreliable information. Capacity limitations. Institutional barriers. Academic culture. Administrative pressure.	Enacting legislation. Amend existing laws (Access to Information and Privacy Act (ATIP) example). Knowledge sharing. Education and training.
Australia					
[57]/2016	Building better research partnerships by understanding how Aboriginal health communities perceive and use data: A semistructured interview study	Occupational engagement: Day-to-day relevance; building professional capacity; emphasise clinical relevance. Trust and assurance: Protecting ownership; confidence in local narratives (story telling); valuing local data sources. Motivation and empowerment: Community engagement; influencing morale about the reasons for data collection and use; reassure and encourage clients about the collection and use of their data. Building research capacity: Using cultural knowledge in culturally appropriate research materials resulting in more accurate data collection and dissemination; promote research aptitude; prioritization of data relevant to professional interests and the interests of the community. Optimising service provision: Data are needed to support sustainable; data are required to guide and improve services; best-practice approaches should be supported. Enhancing usability: The presentation of data should ensure ease of comprehension; improve efficiency of data management; valuing accuracy and accessibility.	Access, use and potential value of clinical and research data.	“Top-down” approaches cannot empower Aboriginal community. Aboriginal people are wary of research. There is a need more Aboriginal involvement and control, over health research practices. Lack of time identified as a major barrier to research training and data use.	Address issues of ineffective data use. Encourage health research that is “strengths-focused”. Support the development of research capacity in Aboriginal communities.
Aotearoa/New Zealand					
[31]/2014	Enacting Kaitiakitanga: Challenges and Complexities in the Governance and Ownership of Rongoā Research Information	Provision of a collective voice for Indigenous rights. Stewardship could be provided by Māori for Māori. Collective control, in contrast to individual control.	Data governance. Health care services.	History of exploitation. Challenges valuing Indigenous knowledges in Western systems of Intellectual Property Rights. Funding limitations. Capacity limitations. International trade agreements that could override exiting governance. Lack of consistency in legal and ethical policies and documents.	Crown resourcing to support the management and governance of Indigenous data. Knowledge repository for traditional knowledge and contemporary research. Partnerships.
[72]/2017	Engaging Māori in biobanking and genomic research: A model for biobanks to guide culturally informed governance, operational, and community engagement activities	He Tangata Kei Tua model Kawa (principles): (i) kia tau te wairua o te tangata (level of comfort), (ii) kia pūmau te mana o te tangata (level of control), and (iii) kia hiki te mauri o te kaupapa (level of integrity). Kaitiakitanga (guardianship). Purpose. Benefit. Respect for Kawa (principles).	Genomics. Biospecimens.	The move of genomics research into clinical practice should work towards clinical benefits and not perpetuate health inequities by excluding populations from sharing in the benefits of genomic medicine advances or creating opportunities for cultural misunderstandings.	Expanding the discussions and presentations of the He Tangata Kei Tua model.
United States					
[20]/2014	Identifying Useful Approaches to the Governance of Indigenous Data	Single-organization data model—outsourcing, Data partnership model—inter-agency coordination, improved efficiency, co-governance of the data asset. Jointly established executive and technical committees to develop and implement framework. Protocols and processes are jointly created. Data commons model—cultivate community of mutual discussion, exchange, and group-sourcing to ensure quality and usefulness. Shared infrastructure or platform that allows members to upload data.	Data governance.	Single-organization data model—what to do with the data belonging to anyone or anything outside of the organization that may be collected and used elsewhere. Data partnership model—Advisory bodies is not data partnership. Data commons model—Near impossible to control the use of data once released into cyberspace.	Integrate disparate, multiple First Nations data sources. Develop and use indicators and performance measures for strategic objectives, visions, and cultural or historical self-understandings of communities. Consolidate information from multiple existing sources (governments and First Nations). Ensure IT infrastructure and technical personnel required to ensure the coordination of data around nations and citizens.
[49]/2014	Exploring pathways to trust: a tribal perspective on data sharing	Partnerships. Development of guidelines that call for re-consent.	Data collection. Data management. Secondary use of research data.	Historical assimilation policies. Bioprospecting.	Partnerships to be situated within the legal framework that protects the sovereign rights of tribal governments. Acknowledgement of IP rights. Protection of Indigenous rights.
[46]/2015	Tribal Archives, Traditional Knowledge, and Local Contexts: Why the “s” Matters	Enabling relationships between Indigenous and non-Indigenous rights holders.	Archival data.	Multiple perspectives, approaches and contexts. Accessibility issues with archival cultural materials in institutions across the globe. Traditional IP system is ineffective.	Repatriation efforts. Development of the Traditional Knowledge (TK) license and label.
[50]/2016	Implementing Qualitative Data Management Plans to Ensure Ethical Standards in Multi-Partner Centres	Community engagement.	Qualitative data. Data protection. Data management.	Institutional Review Board committees cannot anticipate improper data collection, storage, and maintenance.	Invested coordination of qualitative research projects. Inclusion of personnel in Institutional Review Board
[25]/2017	Data as a strategic resource: Self-determination, governance, and the data challenge for indigenous nations in the United States	Existing processes and practices: The development of Indigenous owned and controlled data sets; community-based, nation-driven data governance; assertion of sovereignty over information about Māori; Iwis (tribes) exerting control over the data about their peoples, environments, and businesses; building technical capabilities and partnerships designed to meet tribes’ data needs and support their strategic visions. Emerging processes and practices: Strategically responding to data challenges; engaging with the community to educate leaders and citizens about data; and using data to inform policy decisions and resource allocation that strengthen Indigenous nation sovereignty. Inform internal policy decisions. Identify nation’s assets and allocate resources. Track program and department performance. Access resources. Advocate for external policy changes.	Data. Data Governance.	Inconsistent and irrelevant data. Limited access and utility. Poor quality data. Produced and used within and environment of mistrust. Controlled by those external to the Native nations. Data do not exist to inform tribal needs. Existing data cannot be aggregated in ways meaningful to tribes.	Tribal considerations: Indigenous nation development of institutions to govern data and Indigenous nation engagement of their communities and citizens in defining information needs, designing data collection tools, and interpreting the analyses. Other’s considerations: Acknowledge ID-SOV; include ID-SOV and ID-GOV in tribal, federal, and other governments and organizations’ data policies and processes; invest in capability building to govern data, not just training of individuals to collect and analyse data; and leverage government-to-government relationships between Indigenous nations and other governments to improve data relevance and consistency at federal, state, and other levels. Partnerships.
[17]/2019	Indigenous Data Sovereignty: University Institutional Review Board Policies and Guidelines and Research with American Indian and Alaska Native Communities	Inclusion of Traditional Intellectual Property. American Indian and Alaska Native (AIAN) sovereignty is recognized by the governing body of the state’s universities. Research and institutional engagement principles and best-practice recommendations for collaboration, cultural competency, data storage and sharing.	ID-SOV. Data governance. Institutional governance.	Continue to experience research abuses. Subservience. Struggle to maintain and exercise the right to assert sovereignty in research within community.	Protection and risks to traditional knowledge and intellectual knowledge requires redressing.
[22]/2019	Indigenous data governance: strategies from United States Nations	Data for governance and the governance of data: requirement for accurate, relevant, and timely data for policy and decision making. Additionally requires mechanisms to honour, protect, and control their information both internally and externally. A need to increase capability to govern their data. ➢ Data for governance: Quality, relevance, and access ➢ Governance of data: Ownership and control Utilisation of tribal legislation and tribal research bodies as governance mechanisms in the governance of Indigenous data. Aligning data with Tribal values and visions. Federal investment to support tribal data collection, analysis, and management; tribal authority to integrate federal program funds for comprehensive and streamlined data collection and management efforts; partnerships between federal agencies and tribes to achieve shared data aims; intertribal forums to encourage the exchange of tribal data best practices. Legal requirements including tribal law and Western legal frameworks.	Data. Data governance.	Power differentials within Western data systems continue to disenfranchise Native knowledge systems and Indigenous peoples.	➢ Tribal rights holders Develop tribe-specific data governance principles; develop tribe-specific data governance policies and procedures; generate resources for Indigenous data governance by tribes. ➢ Stakeholders Acknowledge ID-SOV as a global objective; build an ID-SOV framework that specifies the relationships among data processes such as collection, storage, and analysis; create intertribal institutions dedicated to data leadership and building data infrastructure and support for tribes; develop mechanisms to facilitate effective ID-GOV; establish data governance mechanisms that non-tribal governments, organizations, corporations, and researchers can use to support ID-SOV; explore the complexities of individual and collective rights in relation to ID-SOV; explore the relationships among ethics, law, data governance in relation to ID-SOV; grow financial investment in Indigenous data infrastructure and capability; identify common principles of ID-GOV; incorporate ID-SOV rights into all rightsholders’ and stakeholders’ data policies; promote adoption and implementation of common principles of ID-GOV by tribes, governments, organizations, corporations, and researchers within the United States. Recruit and invest in data warriors. Share strategies, resources, and best practices; strengthen domestic and international ID-SOV and ID-SOV connections among Native nations and Indigenous peoples.
More than one region and global					
[2]/2016	Indigenous Data Sovereignty: Toward an agenda	Research should be carried out in partnership with Indigenous peoples. Internal governance and planning. External advocacy. Utilizing and implementing Indigenous governance. Indigenous leadership. Developing capabilities. Developing accountability mechanisms. Code of research ethics. Privacy impact assessments. Partnership (incorporation). Cultural framework. Certification of institutions (OCAP^®^). Legal frameworks (jurisdiction, privacy, repatriation). Co-governance arrangements. IP rights that include cultural and tribal IP. Ethics and resource rights. Council committees that advocate on behalf of Indigenous people. Development of protocols. Data governance is facilitated by tribal sovereignty.	ID-SOV. Data. Human rights.	Lack of reliable data and information. Biopiracy and misuse of traditional knowledge and cultural heritage. Data collection is a political exercise. Definitions and identification of Indigenous peoples. Large gaps in the data and information pertaining to Indigenous people (including environmental, cultural, and social gaps) due to inappropriate or ineffective measures. Denial of Indigenous sovereign governance. Limitation is infrastructure and people capacity.	Governance arrangements that allow for institutional oversight of research and data collections. Developing data governance and capacity with the use of Indigenous data. Exploring the implications of individual vs. collective rights for data linkage, sharing and use. Considerations for what happens with the advancement of “big data” and open data. Data are required for effective governance as well as the effective governance of data.
[38]/2009	Developing a Framework to Guide Genomic Data Sharing and Reciprocal Benefits to Developing Countries and Indigenous Peoples	Consulting with communities (to acknowledge sovereignty and human rights) Complexities of consent (individual, community, and state). Training members of local communities in science and healthcare. Training scientists in how to work with Indigenous and developing communities.	Genomics. Data.	Underdevelopment and colonial exploitation that has resulted in political and economic marginalization. Negative experiences in previous health research, resulting in rejecting genomic research. Scientific abuses.	Formalize an organization to support a long-term effort. Develop resource materials. Develop an information campaign. Launch diplomatic efforts to inform global agencies about the issues. Support internal country discussions and policy initiatives about the issues.
[26]/2017	Indigenous health data and the path to healing	Describes existing principles (Snipp 2016)—1. Indigenous peoples have the power to determine who should be counted among them; 2. Data must reflect the interests and priorities of Indigenous peoples; 3. Tribal communities must not only dictate the content of data collected about them, but also have the power to determine who has access to these data. OCAP^®^ principles supported the governance processes for the use of routinely collected health data at the Institute for Clinical Evaluative Sciences (ICES) in Ontaria, Canada: 1. Access to use of data with Indigenous identifiers are approved by data governance committees organized and population by the relevant Indigenous organizations. 2. Specific application and approval must be sought from the relevant data governance committee before researchers or analysts can access them. 3. Researchers are required to discuss their project with Indigenous community representatives, who may collaborate in the planning, conduct and reporting of the studies. 4. Researchers and staff are to build their capabilities in Indigenous worldviews, research principles, and historical and social contexts. 5. Build the capacity of Indigenous organizations and communities to training Indigenous analysts and epidemiologists. 6. Results are co-interpreted with the communities and the representatives who will decide how the results will be disseminated.	Data. Health reporting.	Results often portray Indigenous health as only a problem and over-emphasize negative findings. There are major gaps in the availability and adequacy of data on Indigenous health.	Greater efforts are needed to track the health of Indigenous peoples. Appropriate governance processes need to be developed through governance and data-sharing agreements.
[15]/2019	Data Management in Health-Related Research Involving Indigenous Communities in the United States and Canada: A Scoping Review	OCAP^®^ provides a valuable framework and rights-based approach to data management. Data management is inclusive of both individual and community rights. Participatory approaches to research. Community engagement.	Data management.	History of unethical and misguided research practices. Concerns have included data collection, interpretation and analysis of data, data security, confidentiality, biospecimens and other data storage, regulatory processes in specimen withdrawal and disposal, data sharing, research dissemination processes.	Community-level governance, including data management terms and practices. Indigenous communities are to participate and develop the policies and protocols guiding data management. A need to better understand the role of data management in shaping research practices to benefit and empower communities. Need for standards for reporting on data management.
[81]/2019	Strengthening the Availability of First Nations Data	Better coordination of First Nations data. Develop a First Nations statistical entity or network. Principles overarching the statistical function—First Nations led; independent; meaningful information; confidential; accessible; First Nations Governance of Data; quality/standardized; partnerships.	Data. Data governance.	Vast amount of data collected on Indigenous peoples. Limitations in accessibility by First Nations to data collected by departments and organizations. Data do not respond to the data needs of First Nations.	Development of First Nations institutions to support statistical capability. A need to address the data needs of First Nations governments and to support the planning, decision making and performance measurement. A need to develop standardized indicators that reflect First Nations and their needs.
[37]/2020	Rights, interests, and expectations: Indigenous perspectives on unrestricted access to genomic data	To support greater diversity and inclusion: 1. Building trust, whereby Indigenous communities decide whether their genomic data and associated metadata are publicly available or accessible on request. 2. Enhancing accountability, in which the provenance of Indigenous samples and genomics data must be transparent, disclosed in publications and maintained with the data. 3. Improving equity, whereby credit should be given to Indigenous communities to support future use and benefit-sharing agreements as appropriate.	Genomics. Data.	Substantial risks, few benefits of genomic research for Indigenous communities.	Agencies need to become responsive to the aspirations of Indigenous communities. Science community to become more sensitive to the concerns of Indigenous communities. Research environment to become more conducive to understanding the cultural implications of genomic research. A need for trust, accountability, and equity.
[21]/2021	Indigenous Data Sovereignty and Policy *	Processes that prioritize Indigenous participation and leadership. Contextual processes and practices. Strategic partnerships. Establishment of tribal data policies. Community engagement. Trust. Centralizing Indigenous priority setting. Accountability. Indigenous control. Disaggregated data. Data used for self-determination. Recognition of existing mechanisms (including treaty and human rights). Alignment with developed ID-SOV principles.	Data. Statistics. Secondary use of data. History. Data governance.	Policy practice lacks the integration of Indigenous worldviews. Statistics do not serve the purposes or interest of Indigenous peoples. UNDRIP is an insufficient foundation for the realization of Indigenous peoples’ rights and interests. Voluntary frameworks and principles may result in limited state commitment to ID-GOV. Limitations in ability for Indigenous peoples to contribute to the policy agenda.	Indigenous-designed legal and regulatory approaches to data founded on ID-SOV principles. Global alliance needed to advocate for and advance a shared vision for ID-SOV. Systematic processes to identify the research with Indigenous data. Access to Indigenous data by Indigenous peoples. Enacting FAIR with CARE.

OCAP = Ownership, Control, Access, Possession; IP [23] = Intellectual Property; ATIP = Access to Information and Privacy Act (Government of Canada 1985a); OCA = Owned, Controlled and Accessible; AIAN—American Indian/Alaskan Native; * Book—with information synthesized from several chapters.

## 4. Findings and Discussion

This systematic review identifies a growing body of literature that provides insights about data governance within Indigenous peoples health research. It shows how the discussion of ID-GOV has evolved since 2001. It demonstrates that there are complex intertwined principles, systems, and processes in ID-GOV. Discussed below is an expansion on the principles, policies, and processes identified by this review.

### 4.1. Indigenous Governance

Indigenous governance was described at the individual- and community-level. This included the importance of valuing and supporting oral traditions and knowledge sharing with the call for the development of legal frameworks and data systems to support Indigenous data [25,31,46]. Values and aspirations as determined through Indigenous governance were identified as being central to the decision-making processes in ID-GOV [2,21,22,37]. Relationships and community engagement to support Indigenous values and aspirations were seen across all 20 publications that referred to governance. Specifically, discussion included enabling relationships and prioritizing relationships between governments and Indigenous communities/nations for the purposes of ID-GOV [25,46].

Engagement processes (in priority setting, research leadership, policy development and advisory roles, as well as data agreements, data protocols and the dissemination of findings) were found to be necessary in identifying and supporting Indigenous practices. These included several different engagement processes including oversight and leadership, advisory roles in conjunction with supporting collaborations, mutual-discussion, and the development of strategic partnerships. The question as to which individuals and communities should provide representative engagement was raised [2] although both individual and collective community engagement were considered necessary for contextual policies, processes and practices in ID-GOV.

Self-determination is a central concept to ID-GOV, mentioned in most publications, whereby concepts of autonomy and the right to make decisions were seen as a requirement in making decisions about data. Decision making incorporated discussions about Indigenous leadership, inter-tribal and cross-community decision making mechanisms, as well as advisory and steering committees. Additionally, nation building, sovereignty and self-determination were mentioned with regards to how data were used for governance [2,21,22]. This concept of decision making is particularly important within data systems because it raises the question of “*who makes decisions on behalf of whom*?” when it comes to Indigenous data.

Several publications described Indigenous or Tribal governments, councils and entities that exercise governance of research and data. This included tribal governments or councils that administer grants and contract and/or engage in research, and data collection activities/surveys [17,25,35,38,47,49,66,70,71]. Specifically, the following examples were seen in the literature:

- Inter-tribal health boards/councils administering and controlling research in their territories [35,49].
- Tribes purchasing their own health information systems [56].
- Tribal archives [17].
- The development of Tribal epidemiology centers [35].
- Developing customized data management systems [19].
- Community controlled organisations designing and collecting population health surveys [82].
- Indigenous peoples and community-controlled organisations having a role in data collection and management [38,57,58,84].
- Developing a First Nations client registry to consolidate demographic information from a variety of health systems [56].
- First Nations communities accessing their community’s data through a web portal, while controlling levels of access to other parties [20].
- First Nations Information Governance Centre’s health surveys and development of OCAP principles to establish new ethical standards for research in First Nations communities [20,30].
- First Nations research center driving partnership/network model of research design and translation, involving community groups, tribal leaders, health planning and service providers [44].
- Indigenous knowledges, principles and theories developed and administered by Indigenous researchers [36,63,64,84].

### 4.2. Insitutional Ethics

Ethical review of Indigenous health research was raised as an underlying feature in data governance within this review. Institutional ethics was generally described as a requirement in Indigenous health research although there were gaps noted in the review process regarding data pertaining to Indigenous people. This denotes that institutional ethical review alone, in its current forms, is insufficient. Ethical review described in the articles generally concerned the ethical policies, processes and/or practices that ordinarily occur in health research. It was thus apparent that institutional ethical review of research that collected and used Indigenous data occurred within western research constructs. Only three eligible publications described Indigenous ethical administration:

- Institutional Review Boards established by Tribal groups in the United States to oversee ethical review of research pertaining to Tribes [15,47].
- Indigenous Tribal groups and councils working with existing ethics review boards to design ethics processes for researchers working in their communities [71].

These publications also described the practices of positioning councils (e.g., Tribal, elders and band) and having advisory committees and/or boards play roles in validating and supporting ethical review [15,47,71]. Importantly, the role of Indigenous ethical administrations, councils and committees directs Indigenous aspirations within ethical review, however, there was limited information on the operationalization of the underpinning policies and processes.

In terms of policies, processes, and practices, ethical review was an important topic for government held administrative data and for genomics data. Individual and community consent and re-consent was raised more than a few times as an area that requires further consideration [38,49]. While data undoubtedly have the potential to provide much needed information to drive nation building and to support Indigenous priorities, it was acknowledged by most articles that some health research has caused harm to Indigenous people and their communities. The benefits (and/or additional harms) from health research were reliant upon how the narrative of the information arising from the data is developed and portrayed [2,21,44,49,57]. The notion of risk was raised in the discussion of genomics data and open data [2,37] as well as the need for the protection of traditional knowledges [17]. Furthermore, it was acknowledged that the different types of data (administrative population level, qualitative, clinical, biomedical samples, genomic, traditional knowledges) as well as data collected and owned by the private sector had different potential ethical implications within the research setting. This is an area that requires more research. The review illuminated the challenges in consistency of ethical review application processes and decisions as well as the capabilities of ethical review boards/committees to provide oversight to support Indigenous data rights in health research.

### 4.3. Socio-Political Dynamics

Socio-political dynamics describe the social and political factors impacting the relationships between Indigenous peoples and governments and/or research institutions. Publications described historical policies and ongoing unequal power distributions due to colonial impacts that had affected relationships between Indigenous peoples and governments, as well as between Indigenous peoples and research institutes. This included the history of exploitation in research as well as bioprospecting of samples resulting in engrained mistrust by Indigenous peoples of research and how research data are used [2,49]. These issues highlight the known unequal power distributions between Indigenous peoples and researchers. It also illuminates the need for governance processes in the use of data that recognize and respect Indigenous values and understandings. To counter this, the requirement for Indigenous people to both own and control the data that pertains to them has become a central point of both ID-SOV and ID-GOV [2,15,21,22,26,30,31,37,44,45,46,49,50,72]. Developing social and human capital to support Indigenous aspirations and to address unequal systems was raised as fundamental to best-practice Indigenous governance of research [44]. Additionally, partnerships and collaborations were described across almost every policy, process, and practice of ID-GOV. The language used to describe partnerships and collaborations can provide insights about the dynamics and decision makers. For example, the “inclusion” of Indigenous people in governance processes implicitly denotes non-Indigenous ownership and control. Historical and ongoing unequal power distributions impacting Indigenous people are well known [85]. It is therefore critical that the dynamics of existing relationships are considered in the development of policies, processes, and practices of ID-GOV. Some governance examples of power-sharing dynamics from the review included:

- Government partnerships to support on-reserve First Nations communities in the planning and development of their health services and models of program delivery, including accreditation and contribution funding for First Nations health services and programs (increasing local involvement in health planning and capacity to plan, deliver and evaluate programs in line with community priorities) [45,77].
- Community involvement in government data collection and evaluation—through governance of information management and evaluation processes [53,59,84].
- Tribes authorizing power for access to certain types of tribal government databases, e.g., sensitive information pertaining to child abuse cases, violent crimes or tribal culture, history, archaeological resources [24].
- Formal Indigenous/tribal resolutions or agreements with universities/research bodies [15,23,44,84].
- Data sharing agreements [18].
- Storage of data at universities with individual depositors setting access conditions [20].
- Multi-institutional genomics platforms [18].
- Indigenous knowledges, theories, relational systems, priority areas incorporated into national initiatives [63].

### 4.4. Data Management and Data Stewardship

Data management describes some of the logistics and implementation policies, processes, and practices of ID-GOV regarding data. Data stewardship on the other hand describes how data will be overseen, including individuals, community and organizational roles and responsibilities. Several facets of data management were identified including data security, data use, data policies and protocols, accessibility and data sharing as well as statistical methods, information systems, data storage and data quality. Additionally, data stewardship facets identified included accountability, responsibility, and legal and regulatory processes. Governance arrangements identified included: the development of data management protocols by Indigenous groups/experts/organisations [19,46,53]; the development of data access protocols [16,26,46,77] and data sharing procedures [49]; the use of Tribal codes/policies [27] as well as Tribal laws to ensure data quality, data storage and security and use [24]; and a governance approach to permissions for access and use of data was also described through a project specific research protocol [50].

Approaches to developing governance procedures in the management and stewardship of data were found to be administrated through Indigenous systems (Tribal Government/Council/community organisations) or non-Indigenous/western research systems. Some examples of Indigenous systems included:

- Data management advisory committees [18,19,25,75].
- Indigenous/First Nations data committees [20,26].
- Indigenous/Tribal research advisory committees [15,16,18,27,70,71].
- Tribal research councils [17,18].
- Community advisory boards [15,57].
- Information planning committees [20].
- Indigenous-led governance board to control and steer direction of National Centre for Indigenous Genomics [29,53,65].
- Indigenous-led governance boards [74].

Legal and regulatory processes specific to data management and stewardship were put forward in the literature. This included discussions on privacy and protection in the disclosure of information, intellectual property laws as well as laws that impact the collection of Indigenous data. Quite a high number of laws were described, although it is not within the scope of this review to describe and discuss them all here. Important to the governance of Indigenous data, there were tensions between internal cultural laws or protocols and external legal systems, particularly regarding the sharing of data within and between nations. This is partly due to intellectual property rights laws interrupting the option of reasonable community control over local materials [46]. Also raised were legal issues concerning the ownership of biomedical samples and data held by institutions under legacy arrangements [29]. West-McGruer described law as a mechanism for the continuity of western conceptions of knowledge [42] which may also raise friction in the development of governance of Indigenous data. Importantly, the right to be counted and the legal structures within nations was also shown to be a continuing area of contention in the collection and quality of Indigenous data [55].

### 4.5. Overarching Influences

Three overarching influences appeared to impact both the discourse and operationalization of ID-GOV. This includes (1) acknowledging and enacting human rights for those nations that have ratified specific declarations and conventions; (2) Capacity, which determines the opportunity to undertake the work that is required in ID-GOV; (3) Funding allocation to support governance development and sustainable systems. Human rights underpin the discourse of enacting ID-SOV and ID-GOV. A number of publications discuss or mention the United Nations Declaration on the Rights of Indigenous Peoples (UNDRIP) [2,17,18,21,22,23,24,25,26,29,31,36,37,38,39,40,41,42,51,59,68,70,71,73,84] as a foundational document in the development of research and also as a tool to support ID-SOV. For example, “ID-SOV is supported by Indigenous peoples’ inherent rights of self-determination and governance over their peoples, country (including lands, waters and sky) and resources as described in the United Nations Declaration on the Rights of Indigenous Peoples” [36]. Further, Indigenous perspectives on access to genomic data [37] mentions Article 31 in a discussion about current discourse surrounding ethics and Indigenous control and rights in genomic research, specifically the inherent right of Indigenous peoples “to maintain, control, protect and develop their cultural heritage, traditional knowledge and traditional cultural expressions, [...] including human and genetic resources” [37].

Building ID-GOV capacity will require developing cultural competence and cultural safety in working with Indigenous populations [17]. It also requires developing the capabilities of Indigenous peoples to collect and use their data for community advocacy and advancement [2,16,21,22,25,26,36,45,57,73,77,81].

### 4.6. Limitations

This review has a range of limitations. Only publications in English were sourced which may result in an under-representation from those counties that publish in other languages. Additionally, due to the heterogeneity of the publications, there were limitations in our ability to appropriately assess the quality of the articles, hence the categorization as publication type so readers are aware of the review process and the publication types where themes and discussion points have arisen.

Other limitations in this emerging field of research include the absence of published information regarding methodological approaches to the development of rigorous yet attainable ID-GOV. Only a handful of articles described governance development and they included limited information on the research processes. We also found evidence that ID-GOV has historically been developed as a part of existing systems without academic publications. For example, since 2008 the Aboriginal and Torres Strait Islander Data Archive protocols have overarching principles to assist best practice in managing the archive in Australia [86]. Furthermore, we only looked at articles published from the year 2001 onwards, which will have missed earlier discussions on the topic which the authors are aware of occurring since the late 1970s.

## 5. Concluding Comments

This review is the most comprehensive assessment of ID-GOV to date. There are a few current, best-practice approaches for researchers that have emerged that could potentially be used in a range of governance strategies moving forward. The review also demonstrates the powerful international congruence of perspectives on ID-GOV.

While there was clear international consensus regarding some definitions and principles used in Indigenous Peoples data governance in health research and reporting, a range of locally developed, unique principles were also observed. Common themes emerged that should be met in the practice of Indigenous health research. There are well advanced country-specific principles that could be adopted or applied to local circumstances. This needs to be done in conjunction with recognising and meeting the needs of local communities. Because of this, global consensus on all facets may not be possible or even desirable because of local needs. The review also identified universally applicable definitions and principles that could support further developments in ID-GOV.

There are several existing ID-GOV frameworks that could be used in practice. There is however limited information on these frameworks were developed. This knowledge may be necessary to advance their utilisation with Indigenous data. Promoting the existence of estabilished frameworks and valorising the development of ID-GOV frameworks that recognise and embed Indigenous ways of knowing, being and doing to researchers, research institutions, governments and communitites will benefit all. This will require moving beyond the western-centrism in health research, which still overwhelmed much of the research included in this review, towards decolonising research and decolonising data. Emerging as an area of further research is an assessment of the quality and effectiveness of data governance frameworks generally and within Indigenous contexts.

There are known and emerging universal themes and actions that can and must be taken to ensure the rights of Indigenous peoples in health research. Without the development of workforce capabilities in ID-GOV, or funding arrangements to support technological and governance infrastructure, Indigenous led ID-GOV will be restricted. It needs to be noted that workforce capabilities in ID-GOV are not exclusive to data. ID-GOV developments must also incorporate recognition and understandings of the socio-political dynamics of Indigenous peoples. This includes approaches that address the historical and contemporary realities in which Indigenous people live, as well as the existing unequal power differentials in society. This is required to enable the aspirations of Indigenous peoples and to minimize the risk of harm from data misuse and misreporting. Incorporating the understandings and learnings from this review of the governance of data in Indigenous health research and reporting provides an opportunity to move towards best-practice approaches in ID-GOV.

## Figures and Tables

**Table 1:** Indigenous Data Governance in health research publication overview.

	N = 68 *n*	% ^
**Year of publication**		
2001–2004	1	1
2005–2008	2	3
2009–2012	4	6
2013–2016	19	28
2017–2020	41	60
2021+	1	1
**Countries/Regions ^1^**		
African nations *	3	4
South Africa	2	2
Australia	12	15
Canada	23	28
Finland	1	1
Aotearoa/New Zealand	11	14
Norway	1	1
United States	17	21
Global **	11	14
**Type of publication**		
Book/Book chapter	5	7
Editorial/Essay	21	30
Original research ^2^	14	21
*Case study*	*3*	*4*
*Cohort study*	*3*	*4*
*Mixed methods*	*1*	*1*
*Qualitative study*	*7*	*10*
Policy	1	1
Report	17	25
Review	10	15

^ Percentages may not add to 100% due to rounding. ^1^ Categories are not mutually exclusive, where publications may include more than one country/region. * African nations include northern African countries. ** Global include 5 or more countries. ^2^ Original research sub-categorized by study design.

## Appendix A

Scopus database search strategy.

ALL ((“indigenous” OR “aborigin* AND torres strait islander*” OR “aborigin* OR torres strait islander*” OR “first nation*” OR “metis” OR “alaskan native*” OR “american indian*” OR “maori*” OR “saami*” OR “inuit*” OR “tribal people*”) AND (“data govern*” OR “data manage*” OR “information manage*” OR “data legislat*” OR “data regulat*” OR “data polic*” OR “data procedure*” OR “data process*” OR “data secur*” OR “data integrity” OR “data steward*” OR “data custodian*” OR “data asset” OR “data sovereign*” OR “data framework*” OR “data compliance” OR “data accountab*” OR “data access” OR “data trust” OR “data ethics” OR “data risk” OR “data disclosure” OR “data reporting” OR “data privacy”) AND (“health research” OR “medical research”)) AND (LIMIT-TO (LANGUAGE, “English”)).

## Appendix B

**Table A1:** Policies, processes and practices as necessary to ID-GOV.

Indigenous Governance	Institutional Ethics	Socio-Political Dynamics	Data Management/Data Stewardship	Overarching Influences
Oral traditions	Benefit vs. harm	Power	Data security and storage	Capacity
Knowledge sharing	Consent	Control	Data analysis and use	Human rights
Values and aspirations	Re-consent	Ownership	Policy/protocols	Funding
Relationships	Risk	Human capital	Data access and sharing	
Community engagement	Data types		Statistics and measurement	
Nation building			Information systems	
Sovereignty			Data quality	
Self-determination			Accountability	
			Responsibility	
			Legal processes	
			Regulatory processes	
